# Determinants of Doctor–Patient Communication in Terms of Patient Rights During the COVID-19 Pandemic

**DOI:** 10.3390/healthcare12212198

**Published:** 2024-11-04

**Authors:** Kamila Jaroń, Mateusz Grajek, Joanna Kobza

**Affiliations:** Department of Public Health, Faculty of Health Sciences in Bytom, Medical University of Silesia in Katowice, Piekarska 18, 41-902 Bytom, Poland; kamila.jaron@gmail.com (K.J.); jkobza@sum.edu.pl (J.K.)

**Keywords:** communication, patient rights, medical staff, health information, coronavirus

## Abstract

Background. Today, the public does not want to be just a passive consumer of health services. Patients often expect to be informed and involved in decisions about their health. With better doctor–patient communication, patients are more likely to follow treatment recommendations. Material and methods. The study was conducted using a face-to-face survey method on a group of 203 adult, independent patients from 2021 to 2022 at a medical facility, i.e., a rehabilitation clinic. Objective. The purpose of this study was to assess the determinants of doctor–patient communication in terms of patient rights. One of patients’ rights is the right to information about their health condition and treatment methods and the right to ask questions when the doctor does not provide details about the treatment or diagnosis or when it is not understandable. Doctors providing information to the patient and the opportunity for the patient to ask questions are key elements in the process of making informed decisions regarding further medical treatment. Therefore, patients were divided into two groups: active (+) and passive in communication (−) with doctors. Results. Patients who were active in communication (33%) wanted to ask questions or had the opportunity to ask the doctor questions, and thus, they were able to take an active part in the discussion with the doctor. In contrast, patients who were passive in communication (67%) did not want to ask questions or did not have the opportunity to ask the doctor questions, and therefore, their active participation in the discussion and thus their right to ask questions may have been limited. The authors’ survey shows that respondents with active communication were significantly more likely than patients with passive communication (almost 100% vs. 86%) to obtain information about their condition (*p* = 0.002), diagnostic methods (*p* = 0.003), therapeutic methods (*p* = 0.00007), treatment results, and prognosis (*p* = 0.0008). Moreover, almost all respondents with active communication as opposed to respondents with passive communication (95% vs. 52%) rated communication with doctors highest (on a scale from 0 to 5), including credible and professional approach to patients (*p* < 0.0001), providing information in clear and simple language (*p* < 0.0001), answering questions asked by patients (*p* < 0.0001), openness and kindness (*p* < 0.0001), maintaining professional confidentiality (*p* < 0.0001), or emotional support (*p* < 0.0001). Conclusions. Hence, the primary key element of the medical consultation is appropriate amount and content of information given to the patient, providing explanations and answering questions. Also importantly, according to the results, active communication between patients and doctors was significantly influenced by female gender, higher education, and a positive evaluation of communication with doctors.

## 1. Introduction

Currently, treating a patient requires a holistic approach that extends beyond addressing the disease [1]. A holistic approach to healthcare includes patient-centered care, aiming to improve patients’ mental and physical health as well as to promote better doctor–patient communication [2]. Good communication promotes patient health through better understanding and adherence to medical recommendations, as well as reduced stress or improved overall patient satisfaction [2,3].

In highly stressful situations, such as sudden pandemics like COVID-19, H1N1, and SARS, creating good doctor–patient communication can be a challenge due to the high patient flow, uncertain situation, and fear of infection [4]. The COVID-19 pandemic undoubtedly forced a rapid evolution in medical practice to adapt to new requirements. At the time, the most important thing was to allow medical personnel to practice safely while maintaining social distance and providing effective clinical care, trying to avoid direct contact with patients whenever possible [5]. During the pandemic, new communication skills were developed and used, which every doctor and patient had to learn and adapt to in a short period of time [5].

The basic principles of practicing good communication can include listening to the patient, empathy, paying attention to the paraverbal and nonverbal elements of communication, and proper information about the nature, course, and prognosis of the disease [1]. Communication problems can result from a lack of attentive listening to the patient, a lack of responsiveness to the patient’s emotions, or barriers to non-verbal communication [6,7]. Doctor–patient communication can also be negatively affected by a lack of mutual respect, reacting to the patient’s emotions, time constraints on visits, a purely medical approach to the patient during the visit that focuses solely on the biological aspects of the disease, disrespectful behavior of the doctor, lack of trust in the doctor, patient dissatisfaction, or a negative assessment of the physician’s attitude [6,7]. It is also important for the patient that the doctor gathering information about him listens carefully, encouraging him to speak without interrupting him or directing the conversation. It is important for medical personnel to constantly improve their efficiency and accuracy in communication and strengthen cooperation between doctors and patients [7]. In the process of educating physicians, little time is devoted to acquiring skills regarding patient–provider communication, and public expectations of healthcare facilities and medical personnel are constantly increasing [8].

Properly conducted communication between patient and medical staff is associated with numerous benefits. Good communication is the basis for a patient’s informed consent and subsequent decision-making during the medical services provided [9]. Information should be given to the patient in “small doses” and the doctor should check that the patient understands it, taking into account possible difficulties with hearing, vision, or memory in elderly people, for example [6]. At the same time, medical personnel play a basic role in the protection, promotion, and implementation of patient rights [10].

The COVID-19 pandemic was an unprecedented shock to healthcare services. Pandemics such as H1N1, SARS, and MERS have occurred primarily outside of Europe, including Mexico, the United States, China, and the Middle East. In Europe, the COVID-19 pandemic caused health systems to face a large-scale challenge for the first time. The rapid spread of the disease took a devastating toll on the entire global economy, as well as causing significant morbidity and mortality among the public. Since March 2020, the COVID-19 pandemic has introduced a number of communication challenges to medical care, which were primarily related to the increased use of personal protective equipment (PPE). Maintaining interpersonal bonds and good communication when providing healthcare in times of pandemic with the use of PPE, especially full-body PPE, has become a new challenge for physicians. The use of PPE obscures facial expressions, muffles voices, can cause difficulty in recognizing and communicating between team members, and can be a barrier to connecting with patients and showing empathy [11].

Research on physician–patient communication during the COVID-19 pandemic presented unique challenges due to the increased use of personal protective equipment. Little of the literature on COVID-19 has focused on the need for attention to the quality of physician–patient communication during a pandemic, which is important regardless of the circumstances. Fear of the spread of COVID-19 and a focus on measures to protect against infection have led to a lack of focus on the quality of doctor–patient communication. Introducing new rules to improve transparency and clarity of communication between doctor and patient is essential for possible future pandemics.

The purpose of this study, conducted during the pandemic, was to assess the determinants of doctor–patient communication in terms of patient rights. Due to the fact that communication problems may also result from providing insufficient information about the treatment and the ability to ask the doctor questions, patients were divided into two groups: active (+) and passive in communication (−). In addition, more specific research questions were also posed to fulfill the purpose of this study:

1. Was the information provided by doctors understandable to patients and conveyed in plain language?
2. How did patients rate the manner of communication, including trustworthiness, professionalism, answering questions, openness and friendliness from doctors, maintaining professional confidentiality, providing sufficient emotional support, or using clear and simple messages?
3. Did the doctors use personal protective equipment?
4. According to the patients, did the doctors spend enough time with them during the examination?
5. Do patients know their rights?

## 2. Materials and Methods

### 2.1. Study Design, Setting, and Participants

The survey form was pilot-tested with a small group of patients to obtain feedback on the ease of understanding the wording of the questions, time to complete the survey, and relevance of the topics. Patient recruitment was carried out at a rehabilitation center to which patients in various departments are referred.

Participation in the study was anonymous and voluntary. Only independent patients, i.e., those who do not need help or care from others, e.g., for nursing, nutrition, or mobility, participated in the survey. Respondents were familiarized with the survey by giving their informed consent to participate. The main criterion for inclusion in the study was being 18 years of age or older and being in good health to take part in the study. No individuals refused to fill in the questionnaires proposed. The study followed the Equator Network reporting guidelines.

### 2.2. Ethical Consideration

The study was conducted in accordance with the provisions of the Declaration of Helsinki and did not require the approval of the Bioethics Committee of the Silesian Medical University in Katowice (decision: PCN/CBN/0052/KB/187/22; 12 July 2022).

### 2.3. Instrument

This study was a cross-sectional study in which authors used a specially designed questionnaire as a data collection method. The first part of the questionnaire consisted of questions about demographics, such as age, gender, and place of residence. The second part consisted of individual factors such as educational level, chronic diseases, hospital wards where respondents stayed, the period of time respondents stayed in a hospital ward, and provinces where patients stayed in hospital wards. The final section consisted of 26 questions relating to communication between patients and medical personnel. The survey consisted of single-choice closed questions. For most of the questions, the authors used a five-point Likert scale to assess the patient’s communication with medical staff, with response options ranging from “definitely yes” to “definitely no”. Communication with physicians was assessed on a scale from 0 to 5, where 0 meant very bad and 5 meant very good. Respondents’ well-being during their stay in the hospital ward was assessed using the World Health Organization’s Five-Point Well-Being Index (WHO-5). The survey questions were formulated based on the patient’s rights under the Law on Patients’ Rights and Patients’ Ombudsman [12]. The time allotted to complete the questionnaire was about 15 min.

### 2.4. Study Procedures

The survey was conducted between November 2021 and March 2022, and the peak incidence of COVID-19 in the fourth wave of the pandemic was in the second half of November 2021; access to hospitals during this time was limited for outsiders. The variable nature of the pandemic meant that there were months when the authors were unable to conduct surveys in hospital wards, so the survey was conducted in one medical facility, i.e., a rehabilitation clinic using a face-to-face survey method. In addition, the communication dynamics in this type of rehabilitation clinic are different from those in hospitals, as patients from different hospital wards are referred to the rehabilitation clinic, making it easier to collect data and evaluate doctor–patient communication after a stay in a hospital ward.

The study sample consisted of 203 adult, independent patients. Patients were divided into two groups: active in communication (+) and passive in communication (−) with doctors. Patients who were active in communication wanted to ask questions, or they had the opportunity to ask questions to the doctor and thus were able to take an active part in the discussion with the medical personnel. Patients who were passive in communication, on the other hand, did not want to ask questions or did not have the opportunity to ask questions to the doctor, and therefore, their active participation in the discussion and thus their right to ask questions may have been limited. Patients active in communication accounted for 33% (*N* = 67) of respondents and patients passive in communication accounted for 67% (*N* = 136) of respondents.

According to the Central Statistical Office, in 2022, there were 6,895,900 people hospitalized in Poland. After calculating the minimum sample size with a confidence level of 95%, a fraction size of 0.9, and a maximum error of 5%, a sample size of 138 people was obtained, which justifies a sample size of 203 as being appropriate to conduct research.

### 2.5. Statistical Analysis

Values of continuous variables were presented as means with standard deviation.

Trait frequencies (qualitative variables) were presented as percentages and N significant. A chi-square test was used to compare trait frequencies across groups/subgroups.

Univariate logistic regression analysis was used to evaluate factors that promote active patient communication.

A multivariate logistic regression model was used to evaluate the determinants of communication with doctors.

Statistical analysis was performed using Statistica version 13.3 (TIBCO Software Inc., Statsoft, Poland).

Program Microsoft Exel was used to collect data.

To assess the internal consistency of the questionnaire, a Cronbach’s alpha test was used for the section assessing doctor–patient communication. The Cronbach’s alpha value was 0.84, indicating high reliability of the tool. This value is within acceptable limits for social surveys, where a value of 0.7 or higher is considered sufficient to establish question consistency. This means that the questions in the questionnaire were internally consistent with each other and measured the same construct, which was the quality of doctor–patient communication.

## 3. Results

The survey included 203 adult, independent patients. Of the respondents, 65% (*N* = 132) were female and 35% (*N* = 71) were male. The average age of the respondents was 55.5 ± 13.7 years (range 19–87). The mean age was 55.0 ± 14.1 years among women and 56.5 ± 13.0 years among men. Men were slightly older, but this difference proved statistically insignificant.

Among the respondents, 25.6% (*N* = 52) had vocational education, 3% (*N* = 6) had primary education, 27% (*N* = 55) had higher education, and the most common group 44.4% (*N* = 90), were respondents with secondary education. Most respondents 53.7% (*N* = 109) resided in a medium-sized city (20–100 thousand residents), followed by 6.9% (*N* = 14) in a rural area, 19.2% (*N* = 39) in a small city (>20 thousand residents), and 20.2% (*N* = 41) in a large city (>100 thousand residents).

Patients were hospitalized in various wards in the Silesian province (98%; *N* = 199), and 2% (*N* = 4) of the respondents were hospitalized in the provinces of Lesser Poland, Opole, Lower Silesia, and Subcarpathia.

About 18% (*N* = 36) of the respondents were treated in the orthopedic ward, 14% (*N* = 29) in the cardiology ward, 14% (*N* = 29) in the gynecology ward, 11% (*N* = 23) in the neurology ward, 9% (*N* = 18) in the general surgery ward, 7% (*N* = 14) on the rehabilitation ward, 6% (*N* = 13) on the internal medicine ward, 6% (*N* = 12) on the urology ward, 3% (*N* = 7) on the pulmonology ward, and 3% (*N* = 7) on the rheumatology ward. There were also single hospitalizations in the ophthalmology, ENT, diabetology, psychiatry, nephrology, endocrinology, dermatology, oncology, and pregnancy pathology departments.

More than half of the respondents, 56% (*N* = 113), were hospitalized due to a planned surgery. In addition, 39% (*N* = 80) of respondents were hospitalized due to a sudden deterioration in health and 4% (*N* = 8) due to rehabilitation. In one case each, the reason for hospitalization was liver disease and risk of premature birth.

Approximately 36% (*N* = 73) were emergency admissions, while 32% (*N* = 65) of patients did not have their hospital date changed. For 30.5% (*N* = 62) of patients, the hospital changed the date of admission (postponing to a later date). In two cases, it accelerated the admission, and in one case, the patient changed the date himself.

Women (76%; *N* = 51) were significantly more likely than men (24%; *N* = 16) to communicate effectively with physicians, X^2^ (1, *N* = 203) = 5.4, *p* = 0.01. In the context of the declared level of education, respondents with a higher level of education communicated better with doctors than respondents with a lower level of education significantly more often, X^2^ (3, *N* = 203) = 22.3, *p* = 0.000006. Analyzing the place of residence, it was observed that respondents who lived in rural areas were significantly more likely to be unable to communicate with doctors than respondents living in urban areas X^2^ (3, *N* = 203) = 17.8, *p* = 0.0004 (Table 1).

### 3.1. Patient’s Understanding of Health Information

**Scheme 1:**
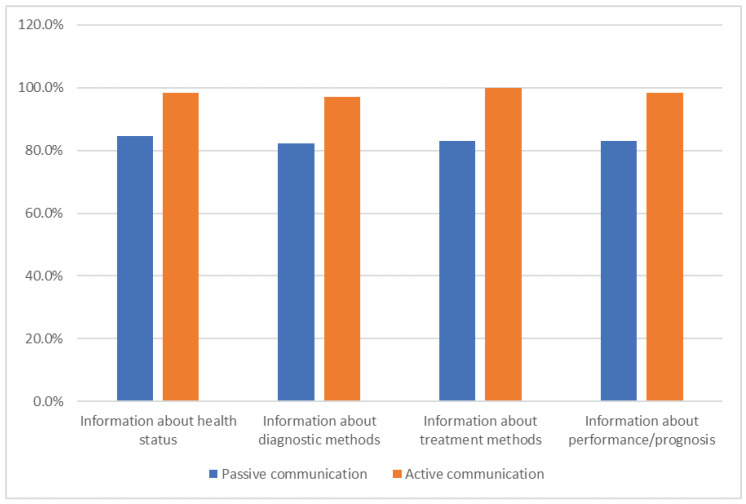
Getting the right information from the doctor with the division into a group with active communication and passive communication with doctors.

The doctors should use procedures related to the protection of patients’ rights, including access to health information, expressing informed consent, and respecting professional confidentiality, intimacy, or dignity [13]. Among the respondents, an analysis was made of whether they were informed about their health condition, proposed and possible diagnostics, therapeutic methods and foreseeable consequences of their use, omission and results of treatment, and prognosis, also taking into account the division of recipients into those with active and passive communication with doctors. The majority of respondents received information about their condition (89.2% (*N* = 181)), diagnostic methods (87.2% (*N* = 177)), treatment methods (88.7% (*N* = 180)), and results and prognosis (88.2% (*N* = 179)). Some people did not receive information about their condition (10.8% (*N* = 22)), diagnostic methods (12.8% (*N* = 26)), treatment methods (11.3% (*N* = 23)), and results and prognosis (11.8% (*N* = 24)). On the other hand, respondents with active communication were significantly more likely than patients with passive communication to obtain information about their health status (98.5% (*N* = 66) vs. 84.5% (*N* = 115), X^2^ Fisher (2, *N* = 203) = 9.03, *p* = 0.002), diagnostic methods (97% (*N* = 65) vs. 82.3% (*N* = 112)), treatment methods (100% (*N* = 67) vs. 83.1% (*N* = 113)), and results and prognosis (98.5% (*N* = 66) vs. 83.1% (*N* = 113)). Successively, X^2^ Fisher (2, *N* = 203) = 8.64, *p* = 0.0003, X^2^ Fisher (2, *N* = 203) = 12.77, *p* = 0.00007, and X^2^ Fisher (2, *N* = 203) = 10.23, *p* = 0.0008 (Table 2 and Scheme 1).

The use of simple and understandable language by medical personnel has been linked to higher quality of communication with patients in healthcare facilities [14]. Among the respondents, about 88.5% (*N* = 179) of all people indicated that the information provided was understandable to them and conveyed in simple language, 1.5% (*N* = 3) of respondents indicated that the information provided was incomprehensible, and 10% (*N* = 21) of people had no opinion on the subject. On the other hand, 98.5% (*N* = 66) among those with active communication and 83% (*N* = 113) of patients with passive communication with doctors felt that the information provided was understandable to them and conveyed in simple language, 2% (*N* = 3) of respondents with passive communication thought the opposite, and 1.5% (*N* = 1) of people with active communication and 15% (*N* = 20) of respondents with passive communication had no opinion on this subject.

**Scheme 2:**
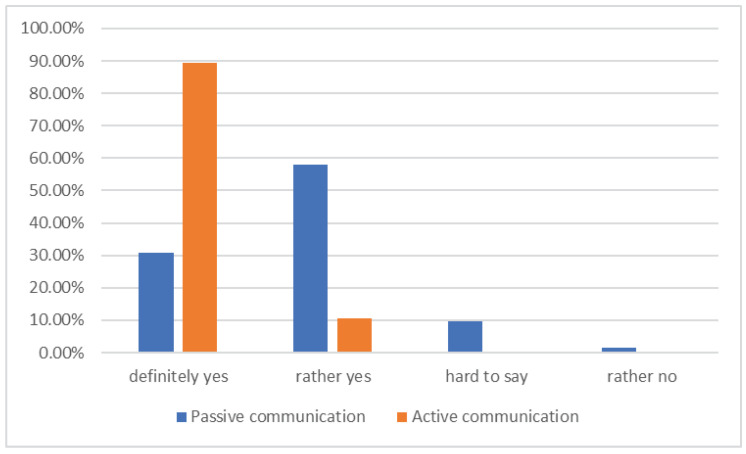
Giving informed consent for medical services with the division into groups with active communication and passive communication with doctors.

In addition, 92.6% (*N* = 188) of respondents indicated that consent for the medical services provided was informed, i.e., preceded by the provision of comprehensive information about the services provided, 1% (*N* = 2) of respondents thought the opposite, and 6.4% (*N* = 13) of people had no opinion on this issue. Patients with active communication with doctors (100% (*N* = 67)) said that their decision was preceded by comprehensive information on the medical service provided to them, which was confirmed by 88.9% (*N* = 121) of patients with passive communication with doctors. On the other hand, “rather not” and “hard to say” were indicated by 1.5% (*N* = 2) and 9.6% (*N* = 13) of patients with passive communication with doctors (Scheme 2).

Respondents rated communication with doctors on a 6-point scale from very bad to very good. A significant proportion of respondents rated the way they communicated with doctors (about 66%) very highly, including the following: trustworthiness, professionalism, comprehensibility and use of simple language, answering questions, characterized by openness and kindness, maintaining professional confidentiality, providing sufficient emotional support. Also, more patients with active communication compared to patients with passive communication rated the way they communicated with doctors very highly (95% vs. 52%).

**Figure 1:**
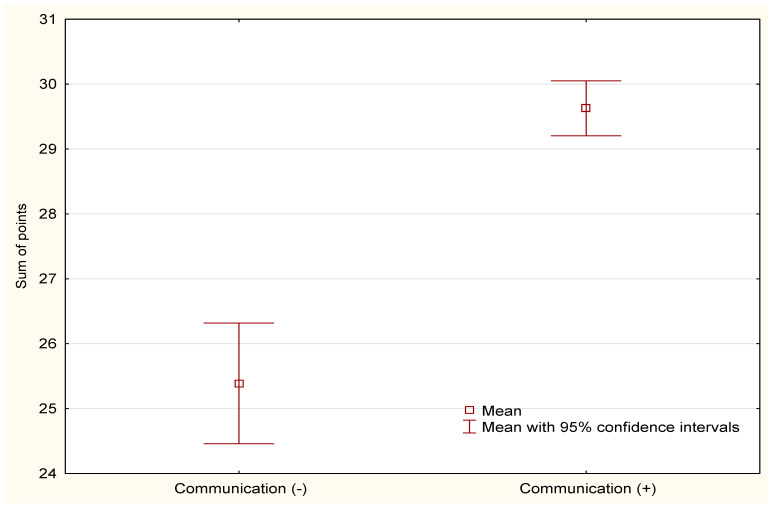
Total scores from the section assessing the way patients communicate with medical personnel, divided into active and passive communication with doctors.

Analyzing the summary evaluation of the means of communication with the doctor’s personnel, patients with active communication rated communication significantly higher (29.5 ± 2.2 (Me: 30)) than patients with passive communication (26.03 ± 5.4 (Me: 30)); U = 22,733; Z = −4.62; *p* < 0.00001 (Figure 1).

### 3.2. Barriers to Effective Communication

Patient rights include the right to respect for dignity, i.e., the right to a benevolent, cultured attitude toward patients by the entity that provides them with medical services [15]. A total of 93% (*N* = 188) of all respondents indicated that they were provided with respect for intimacy and dignity, 1% (*N* = 2) of respondents thought the opposite, and 6% (*N* = 13) of people had no opinion on the subject. In addition, 100% (*N* = 67) of respondents with active communication and 89% (*N* = 121) of patients with passive communication with doctors were provided with respect for dignity and intimacy.

On a daily basis, physicians use surgical face masks, disposable gloves, or hand disinfectant fluids when providing medical services, primarily to protect doctors from infection at a basic level. Of particular importance was the use of personal protective equipment, including masks, visors, and protective suits, during the pandemic due to the high number of COVID-19 infections. The survey found that a total of 95% (*N* = 193) of respondents indicated that medical personnel used personal protective equipment (e.g., mask, visor, apron, protective suit) and 1.5% (*N* = 3) of respondents thought otherwise; 3.5% (*N* = 7) of people had no opinion on the subject. Also, 100% (*N* = 67) of patients with active communication and 92.5% (*N* = 126) of those with passive communication confirmed that medical personnel used personal protective equipment.

Today, doctors spend less and less time with patients during a visit, mainly due to the increasing administrative workload of physicians [16]. A total of 81% (*N* = 165) of all respondents indicated that doctors spent enough time on the examination. However, 6% (*N* = 11) of all respondents thought otherwise, and 13% (*N* = 27) of respondents had no opinion on the subject. Patients with active communication (95.5% (*N* = 64)) said that doctors spent enough time during the examination, while for patients with passive communication, it was 74% of people (*N* = 101).

Knowledge of patient rights is extremely important for all healthcare users. A total of 87% (*N* = 177) of all respondents indicated that they know patient rights, 3% (*N* = 6) of patients thought otherwise, and 10% (*N* = 20) of people had no opinion on the subject. A total of 92.5% (*N* = 62) of patients with active communication and 84.5% (*N* = 115) of respondents with passive communication with doctors indicated that they knew patient rights, but 3% (*N* = 2) of respondents with active communication and 3% (*N* = 4) of patient with passive communication provided negative answers, while 4.5% (*N* = 3) of people with active communication and 12.5% (*N* = 17) of respondents with passive communication had no opinion on the subject.

Respondents were still assessed for well-being during their stay in the hospital ward using the World Health Organization’s Five-Point Well-Being Index (WHO-5). During the study conducted by the authors in the same research group [17] regarding patients’ well-being throughout their stay in a hospital ward, the majority of patients described their well-being as “bad” (78% (*N* = 158)). However, 13% (*N* = 27) of respondents described their well-being as “moderate,” 6% (*N* = 12) of patients described their well-being as “good,” and 3% (*N* = 6) of respondents described their well-being as “very good”. Also, the largest number of patients with active communication (76% (*N* = 51)) and passive communication (79% (*N* = 107)) rated their well-being during their stay in the hospital ward as “bad” (Table 3).

According to the results, active communication between patients and physicians was significantly influenced by female gender, higher education, and positive evaluation of communication with doctors. A high rating of the doctors (>27 points) increased the chance of active communication between patients and doctors by 11 times, female gender increased the chance of active communication with doctors by just over 2.5 times, and higher education increased active communication with doctors by 4 times, while secondary education increased it by less than 2 times and professional education by 1 time (Table 4).

## 4. Discussion

A fundamental element of any relationship is communication, which can be defined as a process of interaction between people, during which information transmitted by appropriate sources can play the role of content related to the health or care of a person [18,19]. The basis for the patient’s interpretation of the information obtained is his knowledge of the essence or course of treatment. A patient who has an elementary knowledge of the actions taken by doctors can more easily assimilate the next, increasingly difficult messages about the type, manner, and purpose of the health services provided [20]. With the above in mind, it is so important for healthcare professionals to explain every detail about the disease, as well as its treatment or the intervention undertaken, because patients may make wrong decisions due to incomplete knowledge [18].

Our own study shows that active communication between patients and physicians was significantly influenced by female gender, higher education, and positive evaluation of communication with physicians. To date, there have been few studies examining the impact of education on patient involvement in decisions about the medical care provided or the relationship between education, health literacy, and active questioning. A study of family physicians and patients in 31 European countries at randomly selected medical facilities found that patients with higher education were more likely to have positive patient–doctor interactions [21]. Another qualitative study involving interviews with 73 men and women with varying levels of education and functional health knowledge living in Australia found that participants from all groups, including those with varying levels of education, believed that the quality of the physician–patient relationship influenced their involvement in medical care. In addition, all participants in the group with higher levels of education reported higher levels of health literacy [22], which in the literature, is defined as the ability to understand, process, and obtain information to make health decisions [23]. Another study conducted in an urban academic center in the northeastern United States on patient visits to hand surgeons’ offices suggests that patients with limited health literacy may benefit from actively engaging in their own medical care and asking questions. Surgeons only occasionally asked patients if they had questions during their visit, but when they did, most patients asked them. Patients with limited health knowledge asked fewer questions about medical care issues than patients with adequate health knowledge because they may have felt more embarrassed or because they may not have understood the information provided by doctors well enough to ask questions and actively engage in medical care. Patients who ask few or no questions may feel reticence, anxiety, and shame admitting that they do not understand something and may also feel less comfortable expressing their concerns. In contrast, patients who had a high level of health literacy and thought their illness was easy to understand asked the doctor the most questions, even though it might seem that because of their understanding of the topic, they would not ask the doctor [23].

According to the prevailing view in the field of legal science and bioethics, decisions made by the patient should be based on the understanding of the information provided by the doctors, and therefore, the messages provided should be conveyed in an accessible manner that is understandable to the person being informed [24]. Failure to provide explanations is typical of the paternalistic type of doctor–patient communication [25].

Patient involvement in informed decision-making is also considered part of the informed consent process [26]. Informed consent for the provision of medical services is a process of continuous dialogue between the doctor and the patient, so the patient’s informed consent depends primarily on the information given by the provider, taking into account the ability to express it and the voluntariness of the decisions [27].

In our own study, the survey conducted by the authors shows that the majority of respondents (about 88%) received information about their condition, diagnostics, treatment methods, results, and prognosis, but as many as 12% of respondents did not receive such information. On the other hand, almost 93% of respondents said that their consent to the medical services provided was informed, that is, preceded by the provision of comprehensive information about the health services provided. After dividing respondents into two groups, those with active and those with passive communication with doctors, the group of those with active communication received almost 100% of information about their health and diagnostic and treatment methods, as well as treatment results and prognosis, and indicated that their consent to the medical services provided was preceded by the provision of comprehensive information in this regard. In contrast, about 86% of respondents with passive communication received information about their health, diagnostic and treatment methods, and treatment results or prognosis and indicated that consent to the medical services provided was informed. In a study conducted at the University Clinical Center in Gdansk, which aimed to compare the opinions of physicians and patients regarding medical communication during hospitalization, 88% of patients positively evaluated the information provided by physicians about their diagnosis and 76% of patients positively evaluated the messages provided regarding the causes of the disease. Of the information regarding possible complications, 72% of recipients of medical services were satisfied and 80% of patients were satisfied with the evaluation of the treatment administered and diagnostic tests recommended [28]. A study conducted at the General Hospital in Modena, Italy, found that patients lacked comprehensive information or insufficient information to give informed consent or make decisions about their healthcare [26]. In another study of patients undergoing major surgery at a military healthcare institution in Nepal, Asia, regarding expressing informed consent by patients, 68.6% of people had the opportunity to ask questions. However, the study found that the facility’s informed consent process did not effectively protect patients’ autonomy, as most did not know the benefits of the study and alternatives to the planned procedure. Shortcomings in the informed consent process included the failure to disseminate adequate information about the nature, duration, advantages, disadvantages, and alternatives to the planned procedure. As the authors of this study point out, shortcomings should be addressed with a well-constructed consent form [29].

COVID-19, as a new disease, is different from other diseases like MERS, SARS, and influenza. Although some diseases may have similar symptoms, coronavirus differed primarily in terms of the speed of spread and severity of the disease. Compared to MERS and SARS, COVID-19 has spread very rapidly, primarily due to increased globalization, and the lack of scientific knowledge about the disease has caused concern from the public worldwide. The most important strategy for inhibiting the spread of the virus is frequent personal hygiene, isolation, maintaining social distance, and wearing masks, which could also affect doctor–patient communication [30]. Our own survey showed that a total of 95% of respondents indicated that medical personnel used personal protective equipment (e.g., mask, visor, apron, protective suit). In addition, 100% of patients with active communication and 92.5% of those with passive communication confirmed that medical personnel used personal protective equipment. Despite this, a significant proportion of respondents rated the way they communicate with doctors (66%) very highly, including the following: trustworthiness, professionalism, comprehensibility and use of simple language, answering questions, characterized by openness and kindness, maintaining professional confidentiality, and providing sufficient emotional support. Also, more patients with active communication compared to patients with passive communication rated the way they communicated with doctors very highly (95% vs. 52%). A study conducted in Italy through interviews among healthcare professionals found that despite the use of personal protective equipment, healthcare workers showed remarkable commitment, creativity, and dedication to patient care. They adapted their communication methods to ensure empathetic and effective interaction with patients despite the physical barriers introduced by PPE. Doctors used eye contact with the goal of also expressing emotion and warmth, increased intonation in their voices, and used badges, as well as using a piece of paper and a pen when necessary to better explain the medical care being implemented. In addition, the use of transparent masks or technology facilitated doctor–patient and patient–family communication [31]. However, according to a study conducted in Chennai, India, using surveys during the pandemic among people staying in hospital, 50% of the surveyed people showed difficulties in doctor–patient communication. Many patients had difficulty communicating with doctors due to physical distancing, use of protective equipment, and limited time with patients due to COVID-19 recommendations [32]. Also, the University of Warsaw’s project to study the humanization of the treatment process and clinical communication between medical staff and patients during the COVID-19 pandemic found that hindered contact with a doctor was most often rated negatively [33].

In addition, a study of patients at two hospitals in Switzerland examined whether patients presenting to the Emergency Department were satisfied with their communication with the doctor. The results indicate that overall, patients were satisfied with communication, but a lower degree of appreciation was observed among younger patients, who were less satisfied compared to older patients [34]. Another study, which aimed to find out the opinions on doctor–patient communication of a selected group of patients at the Clinical Hospital of the University Medical Center in Gdansk, found that all respondents recognized the positive impact of doctor–patient conversation on treatment outcomes [35]. In contrast, in a survey of 26 Italian surgical departments in northern, central, and southern Italy, among outpatients, the surgeons working there received very low scores on actively engaging the patient in asking questions. The lowest scores were achieved in the youngest age group (18–24). The study indicated that the majority of patients undergoing surgery perceived communication as respectful, informative, and understanding, but patients clearly want more active participation in communication [36].

With increasing awareness and education of the public, information on the condition, diagnosis, and therapeutic process should be conveyed in simple, clear, understandable language, avoiding jargon and typical medical vocabulary [37]. The language used by medical personnel is more than just information passed between patients and providers. It has the potential to shape the relationship between the two entities [38]. In the present self-study, 88.5% of all respondents indicated that the information provided was understandable to them and conveyed in simple language. In contrast, 98.5% of those with active communication and 83% of patients with passive communication with doctors felt that the information provided was understandable to them and conveyed in plain language. In a study of plain language practices (a secondary analysis of data from the Partnering Around Cancer Clinical Trials (PACCT) parent study), the authors identified the use of simplified language and recipient-centered definitions as plain language practices used by physicians, among others [14]. In a study of patients interacting with emergency medical teams and medical services operating in hospital emergency departments, using online surveys to assess communication with medical personnel in life-threatening situations, 60.1% of patients tended to agree that providers use understandable language. In addition, respondents indicated that the use of language they understand simultaneously promotes understanding of the procedure being carried out by medical personnel, which increases their sense of safety and has a calming effect on them [37].

Being in a hospital ward can result in a certain degree of restriction of a person’s ability to maintain privacy and dignity, which can be influenced by a variety of factors, including effective communication, the provision of appropriate information by medical personnel, the maintenance of autonomy, or a sense of control [26]. In our own study, 93% of all respondents indicated that they had been provided with respect for intimacy and dignity. Regarding those with active communication, 100% of respondents agreed that they had been assured respect for dignity and intimacy, and for respondents with passive communication, 89% believed so. The study already cited, conducted at the General Hospital in Modena, Italy, also assessed patients’ perceptions of respect for their dignity during hospitalization in the hospital’s surgical wards, and it indicated that dignity was preserved, although not fully to the standards expected by patients. In the study, the most positive responses were given to questions about patient–specialist interactions, which were characterized by kindness, respect, and a warm attitude. Protecting a patient’s dignity can promote not only greater emotional comfort or a sense of well-being but can be an essential prerequisite for recovery [26]. Also, in the aforementioned study involving patients in contact with emergency medical teams and medical services operating in hospital emergency departments, the majority of patients indicated that medical personnel tended to refer to them with respect during the services provided. When asked about respect for the patient’s dignity, most indicated that their dignity was rather respected (56.3%) [37].

Another study, conducted at an academic primary care clinic in Malaysia (Asia), found that reduced time spent with a doctor in outpatient visits can reduce satisfaction among both patients and medical staff [16]. In our own study, a total of 81% of all respondents indicated that doctors spent sufficient time on the examination. Patients with active communication (95.5%) indicated that doctors devoted sufficient time to them during the examination, and the figure was 74% for patients with passive communication. The aforementioned study conducted at osteoporosis clinics in three cities in eastern Poland found that patients most often (43.6%) negatively rated the time spent with the patient by the doctor [39]. In contrast, a study conducted at an academic primary care clinic in Malaysia, Asia, found that patients who perceived the length of consultation time with a doctor during a visit as longer than expected had a significantly higher mean total satisfaction score with the use of medical services compared to patients who perceived the length of consultation as equal to or shorter than expected. This difference between perceived and expected consultation length underscores the importance of medical providers implementing methods to improve a patient’s perception of consultation time with medical personnel, which can simultaneously have an impact on increasing patient satisfaction and enabling the delivery of better-quality healthcare services [16].

The dissatisfaction of some patients with the explanations given by doctors regarding their condition and the failure to devote adequate time to the recipients during the visit may also be due to the large number of duties imposed on doctors and the related susceptibility of individual providers to think and react too hastily when admitting patients [25]. The priority for healthcare providers, regardless of the circumstances, should be to put the patient first during the healthcare activities undertaken and to strive to ensure the best possible communication with them.

## 5. Strengths and Limitation

The study conducted by the authors is innovative due to the limited number of studies on the evaluation of communication between patients and doctors, particularly including a division between those with active and passive communication with doctors. Thus, due to the infrequent topic of the studies conducted in the literature, it was difficult to make comparisons between the results of our own study and previous studies conducted. The study was also limited by conducting the study during the COVID-19 pandemic, staff workload related to the pandemic, and sample size, which was not large enough to present an adequate number of patients communicating with the doctors and receiving treatment. Another major limitation was the study’s focus on stand-alone patients only and its failure to distinguish between COVID-19-infected patients and other patients. Another limitation was the delay between patients’ hospitalization and their responses to the survey. In addition, the study was conducted at a single medical facility, i.e., a rehabilitation clinic, which also did not reflect the communication dynamics that occur during healthcare delivery in other settings, such as emergency departments, where stress and the patient’s condition can affect the quality of communication. Undoubtedly, more research on this topic should be carried out in the country, particularly covering multiple medical settings to increase generalizability. Future studies should look at multicenter studies, especially in more acute care settings (e.g., emergency or intensive care units), to generalize the results across different medical settings. They could combine surveys with interviews or focus groups, which could provide richer qualitative data on why some patients are more active than others during doctor–patient communication.

## 6. Conclusions

The survey found that not all respondents received adequate information about their condition and treatment, which is a basic patient right. Only less than half of the respondents actively cooperated with doctors to clarify information. Respondents who actively communicated with doctors were significantly more likely to receive information about their condition, diagnostic, treatment methods, and prognosis than those who passively communicated, which may have been related to their ability to ask doctors questions. Active communication between patients and doctors was significantly influenced by female gender, higher education, and a positive assessment of communication with doctors. Almost all respondents with active communication but only more than half of the respondents with passive communication rated doctor–patient communication highest (on a scale from 0 to 5), including being credible and professional with patients, providing information in clear and simple language, answering questions asked by patients, being open and friendly, maintaining professional confidentiality, or being emotionally supportive. However, most patients described their well-being during their stay in the hospital ward during the COVID-19 pandemic as “bad”, with a similar result in both groups of patients with active and passive communication with doctors.

Doctors should inform patients that they expect them to ask questions, involve patients more in the opportunity to ask questions and in the decision-making process, and pay more attention to ensuring that the information provided is comprehensive, understandable, and tailored to the audience. It may also be helpful for the patient to make a list of questions before seeing the doctor; in turn, the doctor should check during the conversation that the patient understands the information provided. Medical communication should be tailored to the needs of each patient. To improve and enhance the quality of doctor–patient communication, training should be implemented for doctors, and patients should be more educated about it. When it is necessary to wear personal protective equipment, such as during a pandemic, doctors should pay special attention to the quality of doctor–patient communication, including speaking louder and slower, and using transparent masks or face shields can help with mutual communication. Doctors’ adoption of a patient-centered approach, while remaining flexible during communication challenges that arise, is key to providing patients with the highest quality medical care.

## 7. Practice Implications

Patient-centered care provides the patient with access to information about his or her condition, diagnosis, proposed diagnostic and therapeutic methods, predictable consequences of these methods, and treatment outcomes and prognosis, and it enables healthcare staff to contact the patient, determine his or her needs, and proactively solve problems. This allows recipients to participate in the decision-making process and monitor their treatment. Healthcare communication strategies that include gathering information from patients, engaging patients in defining key messages, checking for understanding, and having physicians use short, tailored, and clear messages should be used regardless of emerging public health crises. However, due to epidemics, more communication training must be provided to physicians to adapt to changing care settings, including ensuring effective communication using protective measures. At the same time, drawing lessons from the pandemic and preparing for possible future epidemics should not be led solely by experts, governments, or politicians. All actions aimed at preventing and combating healthcare crises should also actively engage patients to better understand all aspects of interactions of patients with the healthcare system [4].

## Figures and Tables

**Table 1:** Characteristics of the study group with the division into a group with active communication and passive communication with doctors.

Variable		All Respondents (n; %)	Group		*p*-Value
			Passive Communication n = 136 n; %	Active Communication n = 67 n; %	
Gender	K	132; 65%	81; 59.5%	51; 76%	0.01
	M	71; 35%	55; 40.5%	16; 24%	
Education	Professional	52; 25.6%	46; 34%	6; 9%	0.00006
	Basic	6; 3%	6; 4%	0; 0%	
	Medium	90; 44.4%	57; 42%	33; 49%	
	Higher	55; 27%	27; 20%	28; 42%	
Place of residence	Village	4; 6.9%	10; 7%	4; 6%	0.0004
	City up to 20,000 residents.	39; 19.2%	37; 27%	2; 3%	
	City of 20–100 thousand residents.	109; 53.7%	65; 48%	44; 66%	
	City over 100,000 residents.	41; 20.2%	24; 18%	17; 25%	

**Table 2:** Information about the medical condition, diagnostic methods, therapeutic methods, and treatment results or prognosis with the division into groups with active communication or passive communication with doctors.

Variable		All Respondents (n; %)	Group		*p*-Value
			Passive Communication n = 136 n; %	Active Communication n = 67 n; %	
Receive information about health status	Yes	181; 89.2%	115; 84.5%	66; 98.5%	0.002
	No	22; 10.8%	21; 15.5%	1; 1.5%	
Receive information about diagnostic methods	Yes	177; 87.2%	112; 82.3%	65; 97%	0.003
	No	26; 12.8%	24; 17.7%	2; 3%	
Receive information about treatment methods	Yes	180; 88.7%	113; 83.1%	67; 100%	0.00007
	No	23; 11.3%	23; 16.9%	0; 0%	
Receive information about performance/prognosis	Yes	179; 88.2%	113; 83.1%	66; 98.5%	0.0008
	No	24; 11.8%	23; 16.9%	1; 1.5%	

**Table 3:** Evaluation of patients’ well-being in the hospital ward, with a division into a group with active communication and passive communication with doctors.

Variable:	All Respondents (n; %)	Group	
		Passive Communication n = 136 n; %	Active Communication n = 67 n; %
Wrong well-being	158; 78%	107; 79%	51; 76%
Moderate well-being	27; 13%	15; 11%	12; 18%
Good well-being	12; 6%	8; 6%	3; 4%
Very good well-being	6; 3%	6; 4%	1; 2%

**Table 4:** Factors that foster active communication in the entire study group.

Variable:	Factor or Predictor	Predictor Characteristics	IS (95% PU)
Active communication	Number of hospitalizations	≤8 days	1
		>8 days	1.30 (0.33–3.16)
	Gender	M	1
		K	2.63 (1.15–6.00)
	Place of residence	Village	1
		City	2.38 (0.77–7.33)
	Education	Professional	1
		Medium	1.79 (0.56–5.68)
		Higher	4.10 (1.17–14.28)
	Age	(continuous variable)	0.98 (0.95–1.01)
	Communication with doctors	≤27 points	1
		>27 points	11.14 (2.44–50.70)

## Data Availability

Data are contained within the article and Supplementary Materials.

## Supplementary Materials

The following supporting information can be downloaded at: https://www.mdpi.com/article/10.3390/healthcare12212198/s1. File S1. Questionnaire; File S2. Equator Network.
