# Supplementary material for: Determinants of Doctor–Patient Communication in Terms of Patient Rights During the COVID-19 Pandemic
Source: Healthcare (Basel). 2024 Nov 4;12(21):2198. doi: 10.3390/healthcare12212198 (PMC11545580; doi:10.3390/healthcare12212198)
Supplement: Supplementary file 1 [file healthcare-12-02198-s001.zip › File S1. ANKIETA prawa pacjenta-wersja do druku en(1).pdf]

DEPARTMENT OF PUBLIC HEALTH DEPARTMENT OF PUBLIC HEALTH POLICY DEPARTMENT OF HEALTH SCIENCES IN BYTOM SUM IN KATOWICE

Dear Sirs,

I kindly ask you to participate in a survey on medical staff-patient communication in the context of patient rights during the COVID-19 pandemic. Participation in the survey is voluntary and completely anonymous. The material collected will be used for research purposes only. We kindly ask for your assistance in the survey. The survey consists of 2 parts. The first part of the survey consists of 26 short questions, will take approximately 15-20 minutes to complete and is aimed at hospitalised patients. The second part of the survey is aimed at the patients' relatives and consists of 9 short questions, it will take about 10-15 minutes to complete.

The term 'hospital' as used in the survey includes hospitals and other inpatient treatment facilities.

The term 'COVID-19', as used in the survey, refers to a respiratory disease caused by the SARS-CoV-2 coronavirus.

AGE: ....lat

GENDER: ☐ K ☐ M

EDUCATION:
a) basic
b) professional
c) medium
d) higher

RESIDENCE:
a) village
b) small town with up to 20,000 inhabitants
c) medium-sized city of 20,000-100,000 inhabitants
d) large city with more than 100,000 inhabitants
e) very large city with more than 200,000 inhabitants

YOU ARE A PERSON:
a) independent
b) dependent (needing constant or long-term care and/or assistance from others, e.g. for eating, mobility, personal care)

CHRONIC DISEASES YOU SUFFER FROM
a) cardiovascular diseases (e.g. heart failure, ischaemic heart disease, hypertension)
b) tumours
c) chronic respiratory diseases (e.g. asthma, chronic obstructive pulmonary disease)
d) diabetes
e) osteoporosis
f) chronic kidney disease
g) autoimmune diseases
h) obesity
i) thyroid diseases

j) rheumatoid arthritis
k) neurological diseases
l) not applicable
m) other (which?) ....

HOSPITAL WARD WHERE YOU ARE STAYING
....

VOIVODSHIP WHERE YOU STAYED IN THE HOSPITAL WARD
....

PERIOD WHERE YOU STAYED IN HOSPITAL UNIT (please give approximate time of beginning and end of stay in hospital ward day-month-year)
□□-□□-□□□□ -- □□-□□-□□□□

1) **How was your suspected SARS- CoV-2 coronavirus infection ruled out?**
a) I was tested for SARS- CoV-2 coronavirus after admission to the hospital ward, where I waited for the result
b) I performed a test for SARS- CoV-2 coronavirus on my own
c) I took a test for SARS- CoV-2 coronavirus before entering the hospital premises
d) other (which?) ....

2) **For what reason were you admitted to hospital?**
a) the planned surgery and/or treatment
b) sudden deterioration of health
c) rehabilitation after illness or injury
d) complications of the disease
e) other (which?) ....

3) **Did the hospital change the fixed date of admission?**
a) YES, the date set for hospital admission has been brought forward
b) YES, the set date for hospital admission has been delayed
c) I have independently changed the agreed date of admission to hospital
d) NO, the hospital did not change the scheduled date of admission, I was admitted as planned
e) I have been admitted to hospital on an emergency/urgent basis

4) **Have you been refused admission to hospital?**
a) YES, due to lack of vacancies in the hospital for patients
b) YES, in relation to the exclusion of a ward/hospital from a dedicated treatment activity
c) NO

5) **Were you, on admission to hospital, informed of:**
(please mark an X in the appropriate box):

	**YES**	**NO**
**state of health**		
**the proposed and possible diagnostic methods and the foreseeable consequences of the use/nonuse of the diagnostic methods**		
**the proposed and possible therapeutic methods and the foreseeable consequences of the use/nonuse of the therapeutic methods**		
**treatment outcome/prognosis**		

6) **After receiving the above information (question 5), did you have the opportunity to clarify, to**

ask the doctor about the information provided?
a) definitely yes
b) rather yes
c) difficult to say
d) rather not
e) definitely not

7) Was the information provided understandable to you (e.g. communicated in plain language)?
a) definitely yes
b) rather yes
c) difficult to say
d) rather not
e) definitely not

8) Was your consent for a specific medical service informed, i.e. preceded by the provision of comprehensive information?
a) definitely yes
b) rather yes
c) difficult to say
d) rather not
e) definitely not

9) How would you rate the way you communicate with medical staff? (please adopt a scale of 0-5 where 0- very bad, 5- very good and mark an X in the appropriate box)

	**Medical staff**					
	**0**	**1**	**2**	**3**	**4**	**5**
was credible, professional						
was comprehensible and made use of simplicity of message (plain language)						
responded to the questions asked						
was characterised by openness and friendliness						
has maintained professional secrecy						
provided sufficient emotional support						

10) How would you rate the way you communicate with the nursing staff? (please adopt a scale of 0-5 where 0- very bad, 5- very good and mark an X in the appropriate box)

	**Nursing staff**					
	**0**	**1**	**2**	**3**	**4**	**5**
was credible, professional						
was comprehensible and made use of simplicity of message (plain language)						
responded to the questions asked						
was characterised by openness and friendliness						
has maintained professional secrecy						
provided sufficient emotional support						

11) **During the examination, did the medical staff act in a way that respected your intimacy and dignity (e.g. health services were performed in a separate room or in a sheltered area)?**
a) definitely yes
b) rather yes
c) difficult to say
d) rather not
e) definitely not

12) **Have you had any opportunity to contact (including by telephone) people close to you?**
a) YES, daily
b) YES, once a week
c) YES, less than once a week
d) I have not been in contact with relatives

13) **Did the medical staff assist you in contacting (including by telephone) your relatives?**
a) definitely yes
b) rather yes
c) difficult to say
d) rather not
e) definitely not
f) I did not need help to contact relatives
g) I have not been in contact with relatives

14) **Did the medical staff use personal protective equipment (e.g. mask, visor, apron, protective suit)**
a) definitely yes
b) rather yes
c) difficult to say
d) rather not
e) definitely not

15) **How would you rate the overall safety of the health services provided in the hospital during a pandemic (please adopt a scale of 0-5 where 0- no concern for the welfare and safety of patients, 5- high concern for the welfare and safety of patients)**
☐0 ☐1 ☐2 ☐3 ☐4 ☐5

16) **Do you feel that the medical staff devoted sufficient time to you during the examination?**
a) definitely yes
b) rather yes
c) difficult to say
d) rather not
e) definitely not

17) **Do you feel that the nursing staff devoted sufficient time to you during your activities?**
a) definitely yes
b) rather yes
c) difficult to say
d) rather not
e) definitely not

18) **Have you been vaccinated against COVID- 19?**
a) YES
b) NO

19) Have relatives been able to give you personal items necessary during hospitalisation?
a) definitely yes
b) rather yes
c) difficult to say
d) rather not
e) definitely not

20) Were relatives able to visit you during your stay in the hospital ward?
a) YES, visits by relatives allowed
b) YES, visits by relatives were possible, but only in special situations
c) NO, visits by relatives were banned because of the pandemic
d) I was not visited by people close to me
e) other (which?) ....

21) How would you rate the reduction in visits by relatives of patients staying in hospitals during the pandemic?
a) definitely good
b) rather well
c) difficult to say
d) rather badly
e) definitely wrong
f) there were no restrictions on visits by relatives

22) How would you best describe your well-being during your stay in a hospital ward (please mark an X next to each of the five statements in the appropriate box that best describes your well-being during your stay in a hospital ward, taking a scale of 0-5, where 0- never, 5- all the time)?

	**all the time**	**almost all the time**	**more than half the time**	**less than half the time**	**from time to time**	**never**
**I felt cheerful and in a good mood**	☐5	☐4	☐3	☐2	☐1	☐0
**I felt calm and relaxed**	☐5	☐4	☐3	☐2	☐1	☐0
**I felt active and energetic**	☐5	☐4	☐3	☐2	☐1	☐0
**I woke up feeling refreshed and rested**	☐5	☐4	☐3	☐2	☐1	☐0
**my daily life was filled with things that interested me**	☐5	☐4	☐3	☐2	☐1	☐0

23) For each of the five statements relating to the reading of instructions or leaflets from hospitals or pharmacies, please mark an X in the appropriate box

	**I strongly disagree**	**I rather disagree**	**I have no opinion**	**rather agree**	**strongly agree**

**you find signs in instructions/flyers that you cannot read**					
**the print is too small for you (even though you wear glasses)**					
**the content of the instructions/flyer is too difficult for you to understand**					
**it takes you a long time to read the leaflet/instructions**					
**you need someone to help you read the leaflet/instructions**					

24) For each of the five statements regarding situations when you do not have enough information about your diagnosed disease and its treatment, please mark an X in the appropriate box

	I strongly disagree	I rather disagree	I have no opinion	rather agree	strongly agree
**you gather information on the disease from various sources**					
**you are able to extract the necessary information on the disease**					
**you understand the information you are given regarding your illness**					
**you can give your opinion about your illness to your doctor, your family, your friends**					
**you are able to apply the information you have**					

**learnt about your illness to everyday life**					

25) Please mark an X for each of the four statements concerning obtaining information on the diagnosed illness and its treatment in the appropriate box

	**I strongly disagree**	**I rather disagree**	**I have no opinion**	**rather agree**	**strongly agree**
**you are wondering whether the information you have received about your illness is relevant to you**					
**you wonder whether the information you have received regarding your illness is reliable**					
**you check that the information you have received regarding your illness is up-to-date and reliable**					
**you collect information on your illness to make further healthcare decisions**					

26) In your opinion, do you know your patients' rights?

a) definitely yes
b) rather yes
c) difficult to say
d) rather not
e) definitely not

THANK YOU VERY MUCH FOR FILLING IN THE QUESTIONNAIRE
