# Supplementary material for: Determinants of Doctor–Patient Communication in Terms of Patient Rights During the COVID-19 Pandemic
Source: Healthcare (Basel). 2024 Nov 4;12(21):2198. doi: 10.3390/healthcare12212198 (PMC11545580; doi:10.3390/healthcare12212198)
Supplement: Supplementary file 1 [file healthcare-12-02198-s001.zip › File S2. Equator Network.pdf]

**Title and abstract**	
Title	The topic of the study is the determinants of doctor-patient communication. The study was a cross-sectional survey and consisted of collecting questionnaires among 203 adult, independent patients hospitalized in Poland during the pandemic. The survey questions were formulated based on the patient's rights under the Law on Patients' Rights and Patients' Ombudsman.
Abstract	The survey was conducted from November 2021 to March 2022 at one medical facility, i.e., a rehabilitation clinic. The purpose of this study was to assess the determinants of doctor-patient communication in terms of patient rights. Patients were divided into two groups: active (+) and passive in communication (-) with doctors. Patients who were active in communication wanted to ask questions or had the opportunity to ask the doctor questions, and thus were able to take an active part in the discussion with doctor. In contrast, patients who were passive in communication did not want to ask questions or did not have the opportunity to ask the doctor questions, and therefore their active participation in the discussion and thus their right to ask questions may have been limited. The authors' survey shows that respondents with active communication were significantly more likely than patients with passive communication to obtain information about their condition, diagnostic methods, therapeutic methods, treatment results, prognosis. Moreover, almost all respondents with active communication as opposed to respondents with passive communication rated communication with doctors highest.<br>According to the results, active communication between patients and doctors was significantly influenced by female gender, higher education and a positive evaluation of communication with doctors. A high rating of doctors (>27 points) increased the chance of active communication between patients and doctors by 11 times, female gender increased the chance of active communication with doctors by just over 2.5 times, and higher education increased active communication with doctors by as much as 4 times.
**Introduction**	
Problem formulation	Research on physician-patient communication during the COVID-19 pandemic presented unique challenges due to the increased use of personal protective equipment. Little of the literature on COVID-19 has focused on the need for attention to the quality of physician-patient communication during a pandemic, which is important regardless of the circumstances. Fear of the spread of COVID-19 and a focus on measures to protect against infection have led to a lack of focus on the quality of doctor-patient communication. Introducing new rules to improve transparency and clarity of communication between doctor and patient is essential for possible future pandemics.<br>The study was subjected to aspects of doctor-patient communication based on patient rights.

	Aengst, J; Walker-Stevenson, G.; Harrod, T.; Ivankovic, J.; Neilson, J.; Guise, J-M. Uncomfortable yet necessary: The impact of PPE on communication in emergency medicine. *Int J Qual Health Care*. **2022**, 34(4). doi: 10.1093/intqhc/mzac095.<br>Cvetković, V.M.; Nikolić, N.; Radovanović Nenadić, U.; Öcal, A.; Noji, E.K.; Zečević, M. Preparedness and preventive behaviors for a pandemic disaster caused by COVID-19 in Serbia. *Int J Environ Res Public Health*. **2020,** 9;17(11):4124. doi: 10.3390/ijerph17114124.<br>Bertuol, M.; Barello, S.; Giovanna, C.; Dibennardo, F. Navigating the barriers: an in-depth exploration of how personal protective equipment influences the relational dynamics between healthcare workers and patients. *Acta bio-medica: Atenei Pamensis*. **2024**, 95(2): 1-12. doi:10.23750/abm.v95i2.15671.
Purpose or research question	The purpose of this study was to assess the determinants of doctor-patient communication in terms of patient rights. In addition, more specific research questions were also posed to fulfill the purpose of this study:<br>1. Was the information provided by doctors understandable to patients and conveyed in plain language?<br>2. How did patients rate the manner of communication, including trustworthiness, professionalism, answering questions, characterized by openness and friendliness by doctors, maintaining professional confidentiality, providing sufficient emotional support, or using clear and simple messages by medical?<br>3. Did the doctors use personal protective equipment?<br>4. According to the patients, did the doctors spend enough time with them during the examination?<br>5. Do patients know their rights?
**Methods**	
Qualitative approach and research paradigm	Survey research allows the study of a variety of issues and the use of a multifaceted approach to research. With different types of questions - from closed, to open-ended, scales - surveys allow researchers to obtain a variety of information, which allows for a more comprehensive understanding of the topic under study. However, future studies should look at multicenter studies, especially in more acute care settings (e.g., emergency or intensive care units), to generalize the results across different medical settings and could combine surveys with interviews or focus groups, which could provide richer qualitative data on why some patients are more active than others during doctor-patient communication.
Researcher characteristics and reflexivity	The researchers have a background in medical science, one of the researchers is a dentist. The researchers had no relationship with patients, they were randomly selected for the study, at a rehabilitation center where none of the researchers work. The questions in the survey were based on the patient's rights under the

	Law on Patients' Rights and Patients' Ombudsman. No personal beliefs or characteristics influenced the survey process.
Context	The survey was conducted from November 2021 to March 2022 during the COVID-19 pandemic at one medical facility, i.e., a rehabilitation clinic. The study sample consisted of 203 adult, independent patients. Of the respondents, 65% were female and 35% were male. The average age of the respondents was 55.5±13.7 years (range 19-87). The mean age for women was 55.0±14.1 years, and 56.5±13.0 years among men. Among the respondents, 25.6% had vocational education, 3% had primary education, 27% had higher education, and the most common group 44.4%, were respondents with secondary education. Most respondents 53.7% (N=109) resided in a medium-sized city (20-100 thousand residents).
Sampling strategy	Participation in the study was anonymous, voluntary. Only independent patients, i.e., those who do not need help or care from others, e.g., nursing, nutrition, mobility, participated in the survey. Respondents were familiarized with the survey by giving their informed consent to participate. The main criterion for inclusion in the study was being 18 years of age or older and being in good health to take part in the study. No individuals refused to fill in the questionnaires proposed.<br>According to the Central Statistical Office, in 2022 there were 6 895 900 people hospitalized in Poland, after calculating the minimum sample size with a confidence level of 95%, a fraction size of 0.9 and a maximum error of 5%, a sample size of 138 people was obtained, which justifies that a sample size of 203 is appropriate to conduct research.
Ethical issues pertaining to human subject	The study was conducted in accordance with the provisions of the Declaration of Helsinki and does not require the approval of the Bioethics Committee of the Silesian Medical University in Katowice (decision: PCN/CBN/0052/KB/187/22; 12.07.2022). Informed consent was obtained from all subjects involved in the study. Written informed consent for publication has been waived due to the manuscript does not disclose any participant identifications.
Data collection methods	The survey was conducted in person from November 2021 to March 2022, and the peak incidence of COVID-19 in the fourth wave of the pandemic occurred in the second half of November 2021, access to hospitals at that time was limited for outsiders, the survey was conducted at one medical facility, i.e., a rehabilitation clinic using a face-to-face survey method.
Data collection instruments and technologies	This study was a cross-sectional study, in which authors used a specially designed questionnaire as a data collection method. The first part of the questionnaire consisted of questions about demographics, such as age, gender, place of residence. The second part consisted of individual factors such as educational level, chronic diseases, hospital wards where respondents stayed, the period of time respondents stayed in a hospital ward, provinces

	where patients stayed in hospital wards. The final section, consisting of 26 questions relating to communication between patients and medical personnel. The survey consisted of single-choice closed questions, for most of the questions, the authors used a five-point Likert scale to assess the patient's communication with medical staff, with response options ranging from “definitely yes” to “definitely no”. Communication with physicians was assessed on a scale from 0 to 5, where 0 meant very bad and 5 meant very good. Respondents' well-being during their stay in the hospital ward was assessed using the World Health Organization's Five-Point Well-Being Index (WHO-5). The survey questions were formulated based on the patient's rights under the Law on Patients' Rights and Patients' Ombudsman. The time allotted to complete the questionnaire was about 15 minutes.
Units of study	The study sample consisted of 203 adult, independent patients. Patients were divided into two groups: active in communication (+) and passive in communication (-) with doctors. Patients who were active in communication wanted to ask questions or they had the opportunity to ask questions to the doctor, and thus were able to take an active part in the discussion with the medical personnel. Patients who were passive in communication, on the other hand, did not want to ask questions or did not have the opportunity to ask questions to the doctor, and therefore their active participation in the discussion and thus their right to ask questions may have been limited.
Data processing	Program Microsoft Exel was used to collect data. The raw results of the surveys were entered into a spreadsheet and the data was organized into columns for further study.
Data analysis	Values of continuous variables are presented as means with standard deviation (Figures). Trait frequencies (qualitative variables) were presented as percentages and N significant. A chi-square test was used to compare trait frequencies across groups/subgroups. Univariate logistic regression analysis was used to evaluate factors that promote active patient communication. Statistical analysis was performed using Statistica version 13.3 (TIBCO Software Inc.). To assess the internal consistency of the questionnaire, a Cronbach's alpha test was used for the section assessing doctor-patient communication.
Techniques to enhance trustworthiness	Lengthy time spent in the research environment to better understand the context and avoid superficial interpretations. Discussion of results between authors so that potential errors or ambiguities can be pointed out. Careful sampling to be maximally representative of the study context. Keeping a detailed record of all data analysis processes, allowing verification of how specific conclusions were reached. Also using multiple methods to ensure that conclusions are based on data and not the subjective beliefs of the researcher.
**Results/findings**	
Synthesis and interpretation	The survey showed that not all respondents received adequate information about their condition and

	treatment, which is a basic patient right. Active communication between patients and doctors was significantly influenced by female gender, higher education and positive evaluation of communication with doctors. Doctors should inform patients that they expect to ask questions, involve patients more in the decision-making process and the opportunity to ask questions, and pay more attention to ensuring that the information provided is comprehensive, understandable and tailored to the audience. Medical communication should be tailored to the needs of each patient. In contrast, when it is necessary to wear personal protective equipment, such as during a pandemic, doctors should pay special attention to the quality of doctor-patient communication, including speaking louder and slower, and using transparent masks or face shields can help mutual communication.
Links to empirical data	Questionnaire attached at the end of the manuscript.
**Discussion**	
Integration with prior work, implications, transferability, and contribution(s) to the field	The study conducted by the authors is innovative due to the limited number of studies on the evaluation of communication between patients and doctors, particularly including a division between those with active and passive communication with doctors. Research on physician-patient communication during the COVID-19 pandemic presented unique challenges due to the increased use of personal protective equipment. Little of the literature on COVID-19 has focused on the need for attention to the quality of physician-patient communication during a pandemic, which is important regardless of the circumstances. Fear of the spread of COVID-19 and a focus on measures to protect against infection have led to a lack of focus on the quality of doctor-patient communication. Introducing new rules to improve transparency and clarity of communication between doctor and patient is essential for possible future pandemics. Our own study shows that active communication between patients and physicians was significantly influenced by female gender, higher education and positive evaluation of communication with physicians. To date, there have been few studies examining the impact of education on patient involvement in decisions about the medical care provided or the relationship between education, health literacy and active questioning. In addition, in the authors' own study, the survey conducted by the authors shows that the majority of respondents (about 88%) received information about their condition, diagnostic, treatment methods, results, prognosis, but as many as 12% of respondents did not receive such information.<br>Studies show that it is not uncommon for patients to lack comprehensive information or insufficient information to give informed consent or make decisions about their health care.<br>Despite this, a significant proportion of respondents rated the way they communicate with doctors (66%) very well, including: trustworthiness, professionalism, comprehensibility and use of simple language,

	answering questions, characterized by openness and kindness, maintaining professional confidentiality, providing sufficient emotional support.<br>Most patients were satisfied with communication, but according to other studies, a lower degree of appreciation was observed among younger patients, who were less satisfied compared to older patients. To improve and enhance the quality of doctor-patient communication, training should be implemented for doctors, and patients should be more educated about it, as also indicated by other studies.
Limitations	The study was limited by conducting the study during the COVID-19 pandemic, staff workload related to the pandemic, sample size, which was not large enough to present an adequate number of patients communicating with the doctors and receiving treatment. Another major limitation was the study's focus on stand-alone patients only and its failure to distinguish between COVID-19-infected patients and other patients. Another limitation was the delay between patients' hospitalization and their responses to the survey. In addition, the study was conducted at a single medical facility, i.e., a rehabilitation clinic, which also did not reflect the communication dynamics that occur during health care delivery in other settings, such as emergency departments, where stress and the patient's condition can affect the quality of communication. Undoubtedly, more research on this topic should be carried out in the country, particularly covering multiple medical settings to increase generalizability.
**Other**	
Conflicts of interest	The authors declare no conflict of interest.
Funding	This research recived no external funding.
